# Rapamycin and inulin for booster vaccine response stimulation (RIVASTIM)—rapamycin: study protocol for a randomised, controlled trial of immunosuppression modification with rapamycin to improve SARS-CoV-2 vaccine response in kidney transplant recipients

**DOI:** 10.1186/s13063-022-06634-w

**Published:** 2022-09-15

**Authors:** Matthew Tunbridge, Griffith B. Perkins, Julian Singer, Tania Salehi, Tracey Ying, Branka Grubor-Bauk, Simon Barry, Beatrice Sim, Pravin Hissaria, Steven J. Chadban, P. Toby Coates

**Affiliations:** 1 Central and Northern Adelaide Renal and Transplantation Service (CNARTS), The Royal Adelaide Hospital, Adelaide, South Australia Australia; 2 School of Biological Sciences, The University of Adelaide, Adelaide, South Australia Australia; 3 SA Pathology, Adelaide, South Australia Australia; 4 Department of Renal Medicine, Kidney Centre, Royal Prince Alfred Hospital, Sydney, Australia; 5 Kidney Node, Charles Perkins Centre, University of Sydney, Sydney, Australia; 6 Discipline of Medicine, Adelaide Medical School, The University of Adelaide, Adelaide, South Australia Australia; 7 Viral Immunology Group, Basil Hetzel Institute for Translational Research, Adelaide, South Australia Australia; 8 Molecular Immunology, Robinson Research Institute, University of Adelaide, Adelaide, South Australia Australia; 9 Department of Paediatric Medicine, Women’s and Children’s Hospital, North Adelaide, South Australia Australia; 10 Department of Immunology, The Royal Adelaide Hospital, Adelaide, South Australia Australia

**Keywords:** Kidney transplantation, Randomised controlled trial, Sirolimus, Immunosuppression, SARS-CoV-2, COVID-19, Vaccination

## Abstract

Kidney transplant recipients are at an increased risk of severe COVID-19-associated hospitalisation and death. Vaccination has been a key public health strategy to reduce disease severity and infectivity, but the effectiveness of COVID vaccines is markedly reduced in kidney transplant recipients. Urgent strategies to enhance vaccine efficacy are needed.

**Methods:** RIVASTIM-rapamycin is a multicentre, randomised, controlled trial examining the effect of immunosuppression modification prior to a third dose of COVID-19 vaccine in kidney transplant recipients who have failed to develop protective immunity to a 2-dose COVID-19 vaccine schedule. Participants will be randomised 1:1 to either remain on standard of care immunosuppression with tacrolimus, mycophenolate, and prednisolone (control) or cease mycophenolate and commence sirolimus (intervention) for 4 weeks prior to and following vaccination. The primary outcome is the proportion of participants in each trial arm who develop protective serological neutralisation of live SARS-CoV-2 virus at 4–6 weeks following a third COVID-19 vaccination. Secondary outcomes include SARS-CoV-receptor binding domain IgG, vaccine-specific immune cell populations and responses, and the safety and tolerability of sirolimus switch.

**Discussion:** Immunosuppression modification strategies may improve immunological vaccine response. We hypothesise that substituting the mTOR inhibitor sirolimus for mycophenolate in a triple drug regimen will enhance humoral and cell-mediated responses to COVID vaccination for kidney transplant recipients.

**Trial registration:** Australia New Zealand Clinical Trials Registry ACTRN12621001412820. Registered on 20 October 2021; https://www.anzctr.org.au/Trial/Registration/TrialReview.aspx?id=382891&isReview=true

## Administrative information

Note: the numbers in curly brackets in this protocol refer to SPIRIT checklist item numbers. The order of the items has been modified to group similar items (see http://www.equator-network.org/reporting-guidelines/spirit-2013-statement-defining-standard-protocol-items-for-clinical-trials/).

**Table:** 

Title {1}	Rapamycin and Inulin for booster VAccine response STIMulation (RIVASTIM) - Part 1: The effect of rapamycin on booster COVID-19 vaccine responses in kidney transplant recipients
Trial registration {2a and 2b}	Australia New Zealand Clinical Trials Registry: ACTRN12621001412820. Registered 20^th^ October 2021
Protocol version {3}	3^rd^ October 2021, version 3.0
Funding {4}	No external funding
Author details {5a}	1. Central and Northern Adelaide Renal and Transplantation Service (CNARTS), Royal Adelaide Hospital, Adelaide, SA, Australia 2. Discipline of Medicine, School of Medicine, The University of Adelaide, Adelaide, South Australia, Australia 3. Department of Renal Medicine, Kidney Centre, Royal Prince Alfred Hospital, Sydney, NSW, Australia 4. Kidney Node, Charles Perkins Centre, University of Sydney, Sydney, NSW, Australia
Name and contact information for the trial sponsor {5b}	Central Adelaide Local Health Network Incorporated Royal Adelaide Hospital, Port Road, Adelaide, SA, Australia Principal Investigator: Professor P. Toby H Coates Director of Kidney and Islet Transplantation, Central and Northern Adelaide Renal and Transplantation Service Royal Adelaide Hospital, Adelaide, SA Australia Email: toby.coates@sa.gov.au
Role of sponsor {5c}	RIVASTIM is an investigator-initiated research trial with the coordinating trial center as the study sponsor. The principal and associate investigators are solely responsible for the conception, execution, analysis, and dissemination of the research work.

## Introduction

### Background and rationale {6a}

Kidney transplant recipients (KTRs) are at an increased risk of COVID-19 associated morbidity and mortality, with the combined effects of immunosuppression and prevalent comorbidities contributing to the high rates of adverse outcomes [1, 2]. Meta-analyses have suggested a 28-day mortality approaching 25% for KTRs positive for SARS-CoV-2, and survivors have significant risk of morbidity including hospitalisation, acute kidney injury, and graft loss [2].

The development of effective vaccines which target the SARS-CoV-2 spike protein has been crucial to reducing disease burden and the development severe COVID-19 disease [3]. However, immunocompromised populations such as KTRs were excluded from initial vaccine trials. KTRs exhibit suboptimal vaccine responses and are inadequately protected by current standard two- and three-dose vaccine regimes [4, 5].

To address the inadequate vaccine response observed in KTRs and other immunocompromised groups, additional doses of mRNA vaccine have been recommended. In KTRs, data from a randomised controlled trial suggest that a third mRNA vaccine dose increases the proportion of patients with protective neutralising antibodies to 60%, compared to 25% in the placebo group [6]. While a third vaccine dose improves vaccine immunogenicity, to what extent seropositive patients are protected is unclear, and a significant minority of KTRs fail to seroconvert and therefore require additional strategies [6–8].

Observational data suggests that vaccine responses are significantly affected by immunosuppression regimen. The use of mycophenolate has been identified as a key factor associated with vaccine hypo-responsiveness [5]. Such observations are consistent with earlier reports identifying mycophenolate use as being strongly associated with infection risk among maintenance-phase KTRs, and that use of mycophenolate was associated with inadequate responses to influenza vaccination [9–12]. Conversely, mechanistic target of rapamycin inhibitors (mTORi) have been found to boost vaccine-elicited cytotoxic T cell memory responses in non-human primates, and to improve antibody responses to influenza vaccination in elderly individuals [13, 14]. A recent, multicentre study of over 2000 KTRs reported a 50% reduction in viral infections among KTRs randomised to an mTORi-based regimen, as compared to those receiving mycophenolate, calcineurin inhibitor, and steroid [15, 16].

These observations led to recognition of mTOR complex 1 (mTORC1) activity as a key determinant of the effector versus memory fate decision of antigen-experienced T cells following vaccination [17, 18]. Sirolimus is a potent inhibitor of mTORC1, and the use of mTOR inhibitor-based immunosuppressive protocols has been associated with superior rates of seroconversion, as well as greatly enhanced T cell mediated immunity against SARS-CoV-2 spike protein [19]. Vaccine responses may also be modulated by commensal gastrointestinal microorganisms, collectively the microbiome [20–23]. mTOR inhibitors such as sirolimus have been associated with immunosuppression-regimen-specific changes in the microbiome [24, 25], which may play a role in vaccine immune responses [26].

The RIVASTIM trials are designed to investigate potential strategies to enhance vaccine immunological responses in KTRs using supplementation of the pre-biotic inulin, or immunosuppression regimen alteration. RIVASTIM-Rapamycin is a multicentre, randomised, controlled trial of immunosuppression alteration in KTRs who have failed to develop vaccine-induced protective immunity to COVID-19, prior to a third vaccination. We hypothesise that cessation of mycophenolate and commencing sirolimus in a 3-drug regimen (tacrolimus, sirolimus, prednisolone) will improve the immune response to a third mRNA COVID-19 vaccine.

### Objectives {7}

The primary objective is to compare the proportion of participants in each trial arm who develop protective serological neutralisation of live SARS-CoV-2 virus (ancestral strain) at 4–6 weeks following a third COVID-19 vaccination. The secondary objectives are to determine whether the sirolimus switch (1) improves the proportion of patients that achieve protective anti-receptor binding domain (RBD) IgG antibody, (2) improves the magnitude of vaccine induced T cell response, and (3) is safe and well-tolerated.

### Trial design {8}

RIVASTIM-rapamycin is a multicentre, parallel-arm, randomised, controlled, superiority trial, seeking to examine the effect of ceasing mycophenolate and commencing sirolimus in a 3-drug regimen on the immune response to a third dose of mRNA COVID-19 vaccine in kidney transplant recipients who have failed to demonstrate protective immunity following a two-dose vaccine schedule. KTRs on triple therapy who have received 2-doses of a COVID-19 vaccine will be enrolled and their immune response to vaccination assessed by measurement of serological anti-RBD IgG titre. Those with a satisfactory immune response (anti-RBD IgG ≥ 100 U/mL) will exit the study and be advised to receive a third mRNA COVID-19 vaccination as per recommended guidelines. KTRs who fail to demonstrate protective immunity (anti-RBD IgG <100 U/mL) will proceed to randomisation.

**Fig. 1:**
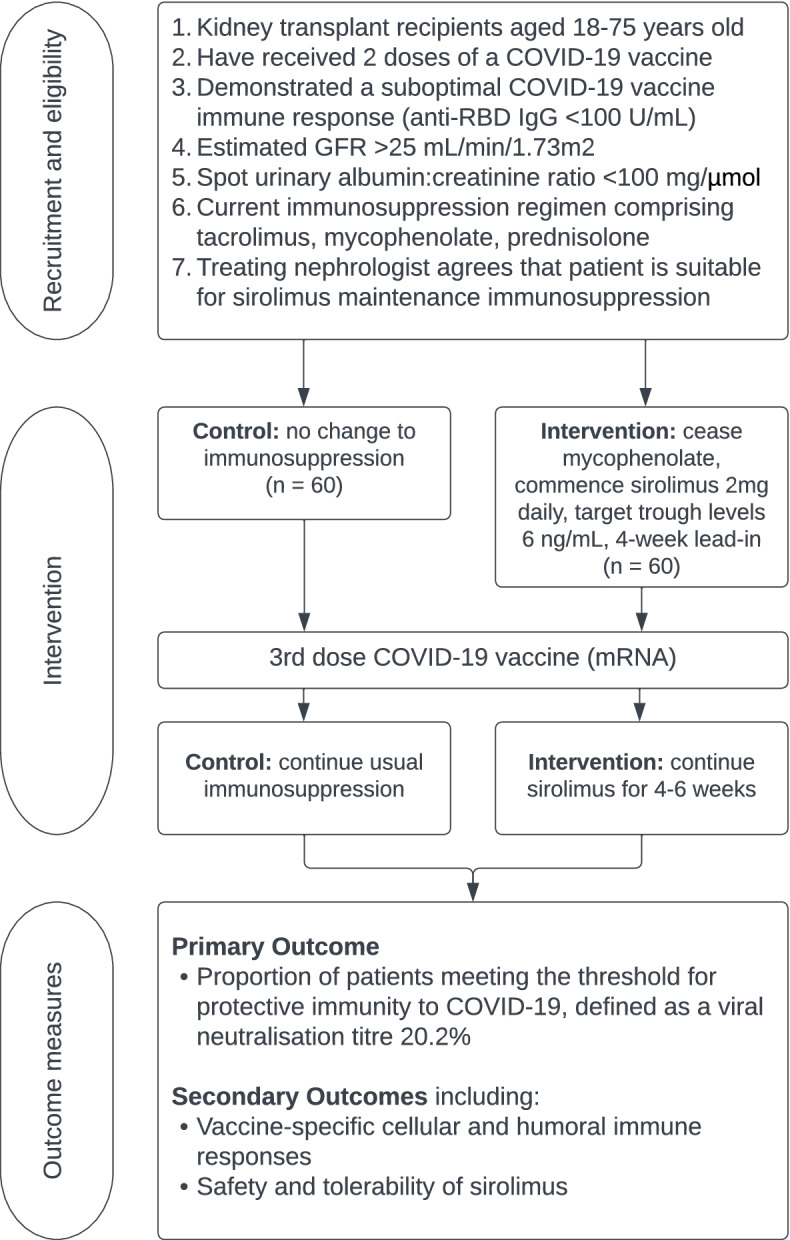
Outline of the trial

Randomisation will occur at a participant level with an allocation ratio of 1:1, stratified by study site and the magnitude of immune response following 2 doses of vaccine (anti-RBD IgG titre; non-responder: < 0.4 U/mL; low responder: 0.4–99 U/mL). An outline of the trial is shown in Fig. 1. Following randomisation patients will either continue their usual immunosuppression regimen of tacrolimus, mycophenolate, and prednisolone (control arm), or have mycophenolate ceased and sirolimus commenced (intervention arm). Sirolimus will be commenced at a standard dose of 2mg daily, with target trough levels of 6 ng/mL and weekly dose titration. Tacrolimus dosage will be adjusted to achieve trough concentration within a range of 3–6 ng/mL. Following a 4-week lead in period, participants will receive a third dose of mRNA COVID-19 vaccine, with the subsequent antibody response measured at 4–6 weeks post vaccination. Participants will continue immunosuppression as per group at least until the time of antibody assessment, and thereafter as determined by their usual nephrologist. The first study participant was enrolled on the 8 November 2021 and recruitment continued through to 15 February 2022, with the final study visit of the last recruited patient in April 2022.

## Methods: participants, interventions, and outcomes

### Study setting {9}

The trial will be conducted at the renal transplant units of two tertiary referral hospitals in Australia: (1) The Royal Adelaide, Hospital, Adelaide, South Australia, and (2) The Royal Prince Alfred Hospital, Sydney, New South Wales.

### Eligibility criteria {10}

The inclusion criteria are:

1. Kidney transplant recipients
2. Aged 18–75 years
3. Estimated GFR >25 mL/min
4. Spot urinary albumin to creatinine ratio <100 mg/μmol
5. Current immunosuppression regimen comprising tacrolimus, mycophenolate, and prednisolone
6. Treating nephrologist agrees that patient is suitable for sirolimus maintenance immunosuppression
7. Have received 2 doses of a COVID-19 vaccine regimen (either adenoviral vector or mRNA-based) and have demonstrably not responded (anti-spike RBD IgG antibody titre below 100 U/mL)

The exclusion criteria are:

1. Aged <18 years or >75 years
2. Significant kidney dysfunction, estimated GFR ≤25 mL/min or spot urinary albumin to creatinine ratio ≥100 mg/μmol
3. Unable or unwilling to provide informed consent to participate in the trial
4. Have received 2 doses of a COVID-19 vaccine regimen (either adenoviral vector or mRNA-based) and have mounted an adequate immune response (anti-spike RBD IgG antibody titre above 100U/mL)
5. Have had documented infection with COVID-19 and/or have detectable SARS-CoV-2 nucleocapsid-specific IgG
6. Known allergy to or intolerance of sirolimus or everolimus

### Who will take informed consent? {26a}

The patient’s treating nephrologist will determine participant eligibility and approach the patient regarding their interest in participating in the clinical trial. Trained research staff will then discuss the trial, provide written information, and seek informed consent during a scheduled clinic appointment. All clinical and research staff involved in consent and enrolment have received trial-specific training and adhere to Good Clinical Practice (GCP) requirements.

### Additional consent provisions for collection and use of participant data and biological specimens {26b}

Participants will provide informed consent to the sampling of biological material (blood and stool), which will be deidentified and stored in the secure research facilities of the participating trial sites and may be used for future studies. Consent for the genomic sequencing of stool microbiota is addressed, with the understanding that no human genomic information will be collected.

### Interventions

#### Explanation for the choice of comparators {6b}

Standard of care immunosuppression for transplant patients consists of a calcineurin inhibitor (CNI, most commonly tacrolimus), an antimetabolite (most commonly mycophenolate), and a corticosteroid (prednisolone/prednisone) [27]. Mycophenolate has been associated with suppressed humoral vaccine response, while sirolimus is associated with improved humoral and cellular vaccine response [19]. There is less risk of acute rejection when replacing mycophenolate with sirolimus, as opposed to replacing CNI with sirolimus [28]. When sirolimus is used in combination with tacrolimus, lower target concentrations of tacrolimus can be used to minimise treatment-related adverse events [28].

#### Intervention description {11a}

Participants will be randomly allocated to their inclusion in one of two groups:

1. Continuation of current immunosuppression regimen including mycophenolate, available in the form of mycophenolate sodium or mycophenolate mofetil
2. Cessation of mycophenolate, and commencement of rapamycin, available as sirolimus (Rapamune®, Pfizer Australia Pty Ltd)

The study products will be provided through the patients’ elected pharmacy. Control arm participants will continue their usual immunosuppression regimen as directed by their treating nephrologist, with standard target trough tacrolimus levels in our institutions ranging from 5 to 8 ng/mL [29]. Intervention arm participants will cease mycophenolate the day prior to commencing sirolimus 2mg daily. Trough drug levels will be taken at days 5–7 with dose titration targeting trough level 6 ng/mL and ongoing weekly levels to ensure stabilisation prior to vaccination.

All trial participants will receive a third “booster” dose of a COVID-19 mRNA vaccine, either Pfizer-BioNTech BNT162b2 (30 μg, IM) or Moderna mRNA-1273 (50 μg, IM) determined by local practice and vaccine availability. Study participants will receive written pre-vaccination information on the benefits and potential risks and harms of the COVID-19 vaccine and be screened for contraindications to immunisation such as serious adverse events attributable to a previous dose of a mRNA COVID-19 vaccine. All patients will be advised of the need to continue with additional public health measures (i.e. physical distancing, hand washing, wearing a face mask, and COVID-19 testing and isolation as required).

#### Criteria for discontinuing or modifying allocated interventions {11b}

Sirolimus has known dose-related adverse effects (AEs). Trough level targets of 6 ng/mL are generally well tolerated [30]. Participants who experience severe adverse events will cease sirolimus and return to their usual immunosuppression regimen. Participants who experience mild AEs related to sirolimus trough levels above target will have their dose reduced. Participants who continue to experience mild AEs (mouth ulcers, peripheral oedema) at target trough level will be offered the option of discontinuing the study intervention and continuing with trial follow-up.

#### Strategies to improve adherence to interventions {11c}

RIVASTIM-rapamycin uses the combination of tacrolimus and sirolimus to facilitate lower target sirolimus and tacrolimus exposure without an increase in the risk of graft rejection [28]. This reduces the likelihood of sirolimus-associated adverse events which are generally dose-related. Following randomisation, participants in the intervention arm will have weekly blood tests for dose titration and to ensure adequate adherence.

### Relevant concomitant care permitted or prohibited during the trial {11d}

All participants will continue with usual transplant management as per local standard of care and at the discretion of their treating nephrologist. Any changes to medications will be recorded. Participants will be asked to continue with their usual diet and medications.

### Provisions for post-trial care {30}

Following completion of the trial intervention, patients will be contacted within 1 week to monitor for adverse events. In conjunction with their treating nephrologist, patients will be offered continuation of the trial drug regimen or return to their previous immunosuppression regimen. Patients converting back to mycophenolate will have this arranged by the trial team, with usual post-transplant care and follow-up then continuing per routine practice under the guidance of the treating nephrologist. The trial sponsor has indemnity to compensate those who suffer from potential harm from as a result of their participation in the research study.

### Management of COVID-19-positive participants during the trial

Study participants who returned a positive COVID-19 result during the trial will be managed in consultation with their treating transplant unit as per local best practice. Participants who contract COVID-19 following randomisation but prior to a third vaccination may have their third vaccine dose delayed. Where possible, participants will be asked to continue with their allocated treatment regimens and attend study visits and follow-up. Positive COVID cases will be excluded from the primary analysis as SARS-CoV-2 infection will confound assessment of the primary outcome measure.

### Outcomes {12}

#### Primary outcome measure

1. The primary outcome is the proportion of participants in each trial arm who develop protective serological neutralisation of live SARS-CoV-2 virus (ancestral strain) at 4–6 weeks following a third COVID-19 vaccination. The protective level is defined as 20.2% of the mean neutralisation level of a standardised cohort of COVID-19 convalescent individuals. This threshold correlates with 50% protection from infection with SARS-CoV-2 (ancestral strain) in the general population [31].

#### Secondary outcome measures

The secondary outcome measures include the following:

2. The proportion of participants in each trial that reach a threshold of serological anti-SARS-CoV-2 (ancestral strain) RBD IgG antibody ≥ 100 units/mL (measured with an Elecsys Anti-SARS-CoV-2 immunoassay [Roche], and equivalent to 100 BAU/mL). This RBD IgG threshold was chosen on the basis of pre-clinical and clinical studies and is consistent with the reported outcomes in published COVID-19 clinical vaccine trials [6].
3. The development of COVID-19 following randomisation, determined by:
  - Positive SARS-CoV-2 PCR test, or rapid antigen test in the setting of symptomatic disease
  - Detection of SARS-CoV-2 anti-nucleocapsid antibodies at the time of primary outcome assessment.
4. Change in the median magnitude of the SARS-CoV-2 spike-specific, antiviral T cell response prior to and at 4–6 weeks following vaccination, determined as the frequency of cells that secrete IFNγ in response to stimulation with spike-protein (ancestral strain)-derived peptides.
5. Phenotypic and functional characterisation of T and B lymphocyte populations
6. Tolerance of sirolimus as determined by drug cessation, drug adherence, and drug-related adverse events including proteinuria, anaemia, leukopenia, rash, mouth ulcers, and pneumonitis.
7. Adverse events following immunisation (AEFI) including adverse events of special interest (AESI) will be assessed via phone consultation at 1 week, and again at 4–6 weeks post-vaccination during the final follow-up visit, and include:
  - Changes in kidney allograft function, determined by serum creatinine, eGFR (CKD-EPI equation), and proteinuria
  - The occurrence of biopsy proven acute allograft rejection
  - The recurrence of primary kidney disease
  - Patient-reported quality of life as recorded by the EQ-5D questionnaire
8. Changes in the community structure, relative abundance, and functional characteristics of the gut microbiome following 4 weeks of sirolimus intervention, determined by 16S-rRNA metagenomic sequencing of participant stool samples.

### Participant timeline {13}

**Fig. 2:**
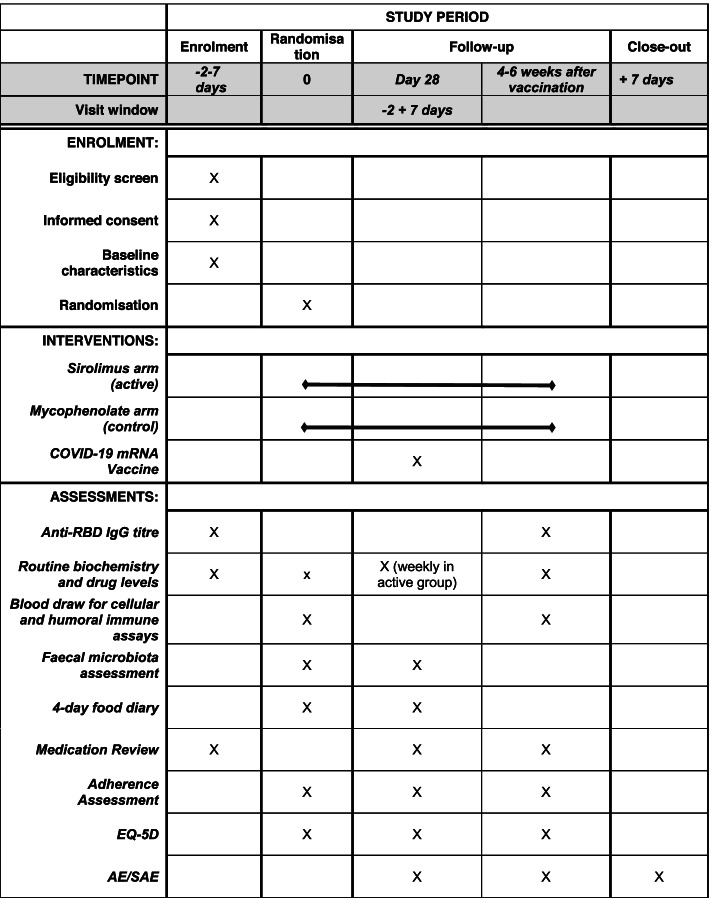
Participant timeline. Standard Protocol Items: Recommendations for Interventional Trials (SPIRIT) checklist. Enrolment, interventions, and assessments. GSRS, gastrointestinal symptom rating scale; EQ-5D, EuroQol five dimensions questionnaire; AE, adverse events; SAE, serious adverse events

Participants are followed from the time of enrolment through until study close-out, 1 week following their final assessment visit. The schedule of enrolment, randomisation, interventions, and assessments is shown in Fig. 2.

### Sample size {14}

This study aims to enrol 120 patients across both sites, with 60 assigned to the sirolimus intervention group and 60 assigned to control. This will provide 80% statistical power (alpha 0.05) to detect an absolute difference of 25% in the proportion of patients who achieve the serological neutralisation titre threshold necessary to provide clinical protection from COVID-19 disease, allowing for a 10% drop-out rate.

### Recruitment {15}

Prospective participants will be identified through the following means:

1. Review of local transplant recipient databases at each trial site. (Only local site clinical staff will have access to identifying information for the purpose of recruitment.)
2. During routine clinical review with their treating nephrologist or transplant centre.
3. Potential participants may have also indicated their interest in trial participation by responding to a QR code displayed during the Transplant Australia COVID Vaccination Update Webinar, broadcast in November 2021.

Potential participants will be approached by their treating nephrologist and offered the opportunity to participate in the trial. Prior to enrolling, patients will be provided with written information regarding the rationale behind the trial, the potential risk and benefits of participation, and the personal commitment involved. Recruitment will continue until target recruitment is fulfilled, or until recruitment of dual-vaccinated transplant recipients is no longer feasible, or if delaying a third vaccination becomes no longer ethically permissible due to clinical urgency. Participants will not receive payment for participation.

## Assignment of interventions: allocation

### Sequence generation {16a}

Participants will be randomised 1:1 to either sirolimus (intervention) or mycophenolate (control). Randomisation will occur via computer-generated stratified block randomisation with randomly permuted block sizes of 2, 4, and 6. Stratification will occur by site and the response to a two dose COVID-19 vaccine schedule (low responder anti-RBD IgG 0.4 - 99 U/mL; or non-responder, anti-RBD IgG < 0.4 U/mL).

### Concealment mechanism {16b}

The allocation sequence is contained and administered centrally through an external web-based randomisation module contained within a purpose-built Research Electronic Data Capture (REDCap) data management platform. The randomisation algorithm and treatment allocation are not accessible to study investigators or research staff.

### Implementation {16c}

The allocation sequence will be generated by an independent and blinded statistician. Trained study investigators will enrol participants, at each study site, and will perform randomisation via the web-based platform and assign the intervention to each participant.

## Assignment of interventions: blinding

### Who will be blinded {17a}

Given the significant changes in prescribed immunosuppression, neither the study participants nor clinical staff will be blinded to treatment allocation. Scientific staff performing the laboratory assays for primary and secondary outcomes will be blinded to patient treatment allocation.

### If blinded, circumstances under which unblinding is permissible, and procedure for revealing a participant’s allocated intervention during the trial {17b}

The design is open label with only outcome assessors being blinded so unblinding will not occur.

## Data collection and management

### Plans for assessment and collection of outcomes {18a}

Trial data are collected prospectively by trial staff and entered on to web-based electronic case record forms (eCRF) maintained on a bespoke REDCap database.

Clinical and laboratory data are collected by study staff from the participant’s electronic medical record. Safety laboratory tests (haematology, chemistry, urinalysis) and enrolment criteria (anti-RBD IgG) are performed at the hospital laboratory of each study site. Blood samples for immunological assessment will be collected by clinical research staff and processed in the on-site immunology laboratory at the Royal Adelaide Hospital. Estimation of participants’ habitual diet will be captured using a 4-day food diary, completed at the time stool samples are collected. Validated questionnaires are completed by participants to capture health-related quality of life (EQ-5D) information.

### Plans to promote participant retention and complete follow-up {18b}

Participants who withdraw from the study, are lost to follow-up, or permanently discontinue the study intervention will be asked to continue with scheduled study visits and follow-up. Outcome data relevant to the trial will be collected from clinical records unless the participants specifically withdraws consent. Reasons for withdrawal, discontinuation, or deviations from the study protocol will be captured in an eCRF.

### Data management {19}

All study data are collected by trained research staff and entered directly onto study-specific electronic data capture forms created and housed within a secure, web-based data management tool (REDCap). The data capture forms contain inbuilt protections to promote data quality, including range checks for numerical data values, restrictions on alphanumeric entries, and prevention of duplicate records. The RIVASTIM REDCap database is stored on secure servers in an on-site limited access data centre at the Royal Prince Alfred Hospital and operated behind the Sydney Local Health District (SLHD) firewall. All electronic information and transmissions are protected via Secure Sockets Layer (SSL) encryption. Access to the RIVASTIM REDCap database is limited to approved research staff, with individual user authentication and logging of all data entry and modification, and access to restricted modules (randomisation, scheduling, and data export) privileged. The database in maintained by the SHLD Information and Communication Technology (ICT) Services with regular back-up processes in place.

### Confidentiality {27}

Prior to study enrolment, participants will consent to research staff accessing their electronic medical record to obtain baseline and demographic information, and the results of laboratory assessments. The privacy and confidentiality of screened and enrolled participants will be preserved with all study data stored in the RIVASTIM REDCap database under a unique numerical study identifier. Access to personal identifying information for participant contact and safety will be limited to trial research staff with privileged access to the REDCap database. Privacy mechanisms within the RIVASTIM REDCap database will remove potential identifiers from data exported for downstream analysis.

No identifying information or individually identifiable participant data will be reported in publications, presentations, or in any report arising from this study.

### Plans for collection, laboratory evaluation, and storage of biological specimens for genetic or molecular analysis in this trial/future use {33}

Blood samples will be taken from participants for immunological assessment at randomisation and at 4–6 weeks following vaccination. Blood will be drawn from participants by clinical research staff and collected in 7 × 9mL lithium heparin and 1 × 8mL CAT serum separator vacutainer tubes. Peripheral blood mononuclear cells (PBMCs) will be isolated from whole blood by density gradient centrifugation in Ficoll-Paque and aliquoted and cryopreserved in liquid nitrogen for batch testing. Sera will be aliquoted and stored at -80°C. Phenotypic and functional assessments of vaccine specific T and B-cell responses will be determined using a variety of laboratory techniques including but not limited to cytometric analysis with intracellular cytokine staining and activation-induced marker (AIM) assays and IFNγ enzyme-linked immunosorbent spot (ELISpot) assays. Using participant serum, the titres of SARS-CoV-2 spike-protein-specific IgG and the capacity of participant serum to neutralise SARS-CoV-2 viral entry into ACE2^+^ cells will be assessed. The capacity of pre- and post-immunisation serum to induce spike-protein-specific antibody-dependent innate immune responses will be measured.

Stool samples will be self-collected by participants using an at home collection kit (OMNIgene GUT OM-200, DNA Genotek, Canada) at baseline and at time of 3^rd^ vaccine dose. Stool samples will be aliquoted and stored at −80°C until batch testing. Analysis of the faecal metagenome will be performed by comparative sequencing of the 16S-rRNA amplicons (V3-V4 region) to identify changes in community structure, relative abundance, and functional characteristics of the gut microbiome.

All biological specimens will be deidentified and labelled with the participants unique study identifier. Stool and blood samples will be stored and maintained in access-restricted laboratory freezers at their corresponding trial site (Adelaide Health and Medical Sciences building, Adelaide, or the Transplant Institute, RPA, Sydney).

## Statistical methods

### Statistical methods for primary and secondary outcomes {20a}

The primary analysis will be by intention-to-treat, with participants assessed according to their treatment allocation. Participants who develop a positive SARS-CoV-2 PCR result during the study will be excluded from the primary analysis to avoid confounding. A per-protocol analysis will also be reported, with participants who failed to adhere or tolerate sirolimus, and participants who withdrew or were lost to follow-up excluded from the analysis. A sensitivity analyses adjusting for potential confounding may be performed should significant imbalances in baseline characteristics between the treatment groups occur.

The primary endpoint is the proportion of patients who achieved a post-intervention serological neutralisation of live SARS-CoV-2 virus (20.2% of the mean neutralisation level of a standardised cohort of COVID-19 convalescent individuals) in both groups using the chi-square test. An unadjusted and adjusted relative risk (RR) will be calculated. For the adjusted RR estimate, the primary outcome of a threshold SARS-CoV-2 serological neutralisation titre will be analysed using a log-binomial regression model. The initial immune response to a two-dose vaccine schedule (anti-RBD IgG titre; low responder: 0.4–99 U/mL; or non-responder: <0.4 U/mL) will be included in the model as a fixed effect, with study site as a random effect.

Secondary outcomes will be analysed using univariate and multivariate methods dependant on the outcome type. Baseline characteristics and demographic data will be reported as mean ± SD for normally distributed data and median ± IQR for non-normally distributed data, with categorical variables reported as frequencies. All statistical analyses will be described in detail with arising publications. A two-sided significance level of 5% will be used for all analyses.

### Interim analyses {21b}

No interim analyses are planned.

### Methods for additional analyses (e.g. subgroup analyses) {20b}

Subgroup analyses will be performed to examine for statistical interaction between the treatment arm and (1) initial response to 2-dose vaccine schedule (non-responder or low-responder) and (2) duration between previous vaccine dose (less than, or greater than 6 weeks) and randomisation. Patients who develop primary COVID-19 infection during the study period will have both primary and secondary outcomes analysed as a pre-specified subgroup analysis.

### Methods in analysis to handle protocol non-adherence and any statistical methods to handle missing data {20c}

A per-protocol analysis will also be reported, with participants who failed to adhere or tolerate sirolimus, and participants who withdrew or were lost to follow-up excluded from the analysis. Multiple imputation will be used to handle data missing at random from baseline characteristics. Data missing at random for the primary and secondary outcome will not be imputed, with these cases excluded from ITT analysis. If > 10% of the primary outcome data is determined to be missing not at random, a best-worst and worst-best case sensitivity analyses will be performed.

### Plans to give access to the full protocol, participant-level data, and statistical code {31c}

The complete trial protocol and statistical code used for analyses will be made publicly available following publication of the primary results. Following publication of all study results, deidentified participant-level data may be made available upon reasonable request to the principal investigator, or in the case of published works, through the corresponding author.

## Oversight and monitoring

### Composition of the coordinating centre and trial steering committee {5d}

The coordinating trial centre is located at the Royal Adelaide Hospital. The Trial Steering committee (TSC) is co-chaired by the Principal Investigator (PI) at each study site and includes the trial associate investigators. The TSC is responsible for the study conception, drafting, and completion of the study protocol and associated documents, recruitment plan, data monitoring and integrity, end point adjudication, and approving publications arising from the study.

### Composition of the data monitoring committee, its role and reporting structure {21a}

A data safety monitoring board has not been established and was not warranted in this study, given the short duration of the intervention and follow-up. The TSC is responsible for the scientific integrity of the trial and will monitor safety and operational data and will fulfil reporting obligations to the trial sponsor.

### Adverse event reporting and harms {22}

All protocol deviations and AEs will be documented, regardless of their potential relationship to the study intervention. AEs will be recorded using an adaptation of the National Institute of Health’s Common Terminology Criteria for Adverse Events by a study team member on an eCRF. Recorded information on each AE will include a descript of the AE, the onset date, duration, the resolution of the AE, the severity and seriousness, any action taken as a result of the AE, the outcome of the AE, and the likelihood of the relationship of the AE to a study intervention. Screening for adverse events will occur during each study visit and during scheduled clinical follow-up with their treating nephrologist and will be captured up to 7 days following the final study visit. Adverse events following immunisation (AEFIs) with the exception of mild and/or short-lived symptoms will be reported to the Therapeutic Goods Administration (TGA). Serious adverse events (SAEs) will be reported to the trial sponsor with 24-h of the study team being made aware of the event.

### Frequency and plans for auditing trial conduct {23}

There are no plans for trial audit given the short duration of the trial intervention and follow-up.

### Plans for communicating important protocol amendments to relevant parties (e.g. trial participants, ethical committees) {25}

Amendments to the study protocol will be approved by the human research ethics committee (HREC) at the coordinating trial centre, followed by local site governance prior to implementation. The trial registration information contained with the Australia New Zealand Clinical Trial Registry (ANZCTR) will be updated with any protocol modifications.

## Dissemination plans {31a}

The results of the RIVASTIM-inulin trial will be published in peer-reviewed academic journals and presented at national and international scientific meetings. Additionally, a lay summary containing the study aim, salient findings, conclusions, and a take home message will be prepared and distributed to trial participants, research staff, and interested members of the transplant community. The lay summary will be distributed via direct approaches to trial participants and be made widely available through electronic media including newsletters, social media, and websites.

## Discussion

Interventions to improve COVID-19 vaccine responses in transplants recipients are required. Preclinical studies and observational findings in KTRs suggest that the mTOR inhibitor sirolimus may enable enhanced immunological responses to COVID-19 vaccination, as compared to standard of care immunosuppression which includes mycophenolate.

This multi-centre, prospective, randomised, controlled trial has been designed to measure the effect of temporarily modifying maintenance immunosuppression with sirolimus, in conjunction with withdrawal of mycophenolate, on correlates of immune protection from COVID-19. The trial uses a pragmatic, established immunosuppression protocol to boost vaccine responses. Sirolimus is a commonly used immunosuppressant that is well tolerated at trough target levels of 3–6 ng/mL. As such, improved immunity in the treatment arm would provide strong justification for the use of sirolimus switch to enhance vaccine-induced protection against COVID-19 among KTRs.

There are limitations to a rapidly designed trial in the context of the COVID-19 pandemic. At the commencement of the trial, Australia had a largely SARVS-CoV2-naïve population with minimal community transmission. However, with the easing of social restrictions and border controls there is increasing community prevalence. Thus, trial recruitment may be limited by the prerogative to not delay booster vaccination in a vulnerable population, and in the loss of potential recruits to either having already had booster vaccination or having developed COVID-19. Additionally, while this study will provide a targeted strategy for immunosuppression modification, it will not be possible to discern the individual contributions of withdrawing mycophenolate versus addition of sirolimus on the immune response.

### Trial status

**Table:** 

Protocol version	3.0
Protocol date	3 October 2021
Recruitment start date	8 November 2021
Anticipated recruitment end date	15 February 2022
Reason for submission after recruitment cessation	Early recruitment closure before submission as unable to enrol target 120 patients due to (1) reducing number of eligible participants with ongoing vaccine roll-out and (2) increasing community prevalence of COVID-19 making further delays in vaccination due to trial participation unacceptable

## Data Availability

Datasets and results generated as part of this study will be jointly owned by Central Adelaide Local Health Network, the University of Adelaide, and the Royal Prince Alfred Hospital (RPA, SLHD). Deidentified participant data may be made available from the corresponding author of published works upon reasonable request and submission of a research plan of appropriate scientific merit and ethical standing.

## Abbreviations

AEFI: Adverse events following immunisation
AEs: Adverse events
ANZCTR: Australia and New Zealand Clinical Trials Registry
CALHN: Central Adelaide Local Health Network
CKD-EPI: Chronic Kidney Disease - Epidemiology Collaboration
eCRF: Electronic case report form
eGFR: Estimated glomerular filtration rate
EQ-5D: EuroQol five dimensions
GCP: Good Clinical Practice
GSRS: Gastrointestinal symptom rating scale
HREC: Human research ethics committee
KTRs: Kidney transplant recipients
mTOR: Mechanistic target of rapamycin
mTORi: Mechanistic target of rapamycin inhibitor
mTORC1: Mechanistic target of rapamycin complex 1
RBD: Receptor-binding domain
REDCap: Research Electronic Data Capture
RIVASTIM: Rapamycin and inulin for booster vaccine response stimulation
SAEs: Serious adverse events
SARS-CoV-2: severe acute respiratory syndrome coronavirus 2
SCFA: Short-chain fatty acids
SLHD: Sydney Local Health District
TGA: Therapeutic Goods Administration
TSC: Trial steering committee
